# The COVID-19 pandemic and critical laboratory functions. Can fast-track molecular testing reduce work absence in the laboratory?

**DOI:** 10.1177/17571774251330455

**Published:** 2025-04-03

**Authors:** Thea A Andersen, Johan Bjerner, Trygve Tjade, Trond E Ranheim, Eyvind W Axelsen, Michael Sovershaev, Ying Chen, Peter Gaustad

**Affiliations:** 1 Fürst Medical Laboratory, Oslo, Norway; 2 6305 The Faculty of Informatics and Natural Sciences, Institute of Informatics, University of Oslo, Oslo, Norway; 3 158935 Faculty of Health Sciences, Institute of Life Sciences and Health, Oslo Metropolitan University, Oslo, Norway; 4 Faculty of Medicine, Institute of Clinical Medicine, University of Oslo, Oslo, Norway

**Keywords:** Infection prevention and control practices, fast-track molecular testing, intervention, laboratory staff, infection control, COVID-19, SARS-CoV-2, medical laboratory

## Abstract

**Background:**

Amid the SARS-CoV-2 pandemic, laboratories faced the challenge of maintaining diagnostic operations while adhering to infection prevention and control (IPC) guidelines. We investigated the impact of implementing rapid molecular testing of employees of a large medical laboratory to prevent workplace transmission.

**Aim/objective:**

To evaluate if fast-track PCR diagnostics, alongside local infection control measures, could reduce internal transmission and workplace sickness absence.

**Methods:**

Employees with respiratory symptoms, but testing negative for SARS-CoV-2, were allowed to work if clinically healthy. All included employees completed a questionnaire and underwent SARS-CoV-2 antibody testing post-pandemic. Data on sickness absence were retrieved from local human resources systems, and comparative analyses were conducted between the pre-pandemic and pandemic periods.

**Findings/results:**

Of 153 participants, 84 (55%) reported having had COVID-19, with 12 (14%) suspecting workplace transmission. Six (4%) tested positive for SARS-CoV-2 IgG nucleocapsid despite no COVID-19 diagnosis. Among 101 (66%) reporting respiratory symptoms and negative SARS-CoV-2 tests, 80 (79%) were allowed to return to the workplace. Mean workplace sickness absence during the pandemic 2020 (3.74%) and 2021 (4.19%) was significant lower compared with sickness absence in the laboratory before the pandemic in 2019 (4.54%). No larger outbreaks in the laboratory were recorded.

**Discussion:**

SARS-CoV-2 infections in the laboratory were mostly symptomatic and acquired outside the workplace. The combination of local IPC and rapid and frequent testing of employees facilitated an effective infection control and minimized workplace absence, maintain diagnostic operations.

## Background

SARS-CoV-2 was identified in January 2020, with WHO declaring a global pandemic on March 11, 2020 (Jin et al., 2020). Norway reported its first case on February 26th, 2020. Norway implemented Infection prevention and control (IPC) measures from March 12, 2020, including hand hygiene, cough etiquette, protective equipment, social distancing, PCR testing, isolation, quarantine, and contact tracing (Bruun and Lange, 2023). Such IPC have long been known to prevent spread of infection, and they are used on a large scale in healthcare worldwide (Strich and Palmore, 2017; Wilder-Smith et al., 2020). Asymptomatic courses of COVID-19 have been observed, providing a risk of infection spread (Kronbichler et al., 2020; Ma et al., 2021). Other cold viruses may have overlapping symptoms with SARS-CoV-2 (Czubak et al., 2021; Lippi, 2020). The national preparedness plan against outbreaks of serious infectious diseases emphasizes rapid diagnosis of suspected cases and isolation of infected individuals (Action plan for better infection control with the aim of reducing healthcare-associated infections 2019-2023, 2023). National guidelines during the COVID-19 pandemic recommended testing all symptomatic individuals with a subsequent high workload for Norwegian medical microbiological laboratories with no opportunity to work remotely. Due to reagent shortages, high sample numbers, and lack of qualified personnel, test turn-around time increased to 3 days or more (Kanestrøm et al., 2021). The main goal of this project was to evaluate, given local IPC implemented, the safety of allowing laboratory staff to return to the workplace after a negative SARS-CoV-2 PCR test. In addition, two sub-goals were investigated: The number of employees testing positive for COVID-19 infection and possible route of infection, and the number of employees with an asymptomatic infection.

## Methods

### Study population

Fürst Medical Laboratory has approximately 400 employees and its main facility is in Oslo, along with three small sampling stations. During the pandemic, a streamlined testing protocol was implemented in accordance with health authority IPC guidelines. The employer provided SARS-CoV-2 fast-track testing to employees experiencing respiratory symptoms or those who had close contact with a confirmed COVID-19 case. Only those who tested negative and were clinically healthy were permitted to return to work.

Participation in the study was voluntary, and an invitation to participate was extended to all employees (*N* = 437). The gender distribution of employees was 365 women and 72 men. The exclusion criterion was absence from the workplace for more than 2 months (due to home office, pregnancy, long-term sick leave, and other extended leaves), as they needed to be affected by workplace IPC measures. The included employees participated in the study between 13.03.2020 and 29.04.2022. Serum samples from 202 participants were analyzed. Of these, 45 were excluded for not submitting the questionnaire and 4 due to extended absence. After removal of those participants, 153 subjects remained enrolled in the study. A total of 133 women and 20 men participated, and the majority age was 41–60 years.

### PCR methods

Nasopharyngeal or throat swabs were collected from employees with respiratory symptoms. Samples were analyzed 1–2 times daily, with test results available within 30 minutes (ID NOW™, Abbott Norge AS) to a maximum of 8 hours (RIDA®GENE, R-Biopharm, Montebello Diagnostics). Swabs were collected using Copan eSwab on recommended transport medium from the manufacturer. Starting from December 2021, ID NOW™ from Abbott Diagnostics Scarborough (Abbott Diagnostics, 2024) was used alongside DirectDetect™ SARS-CoV-2. ID NOW™ is a point-of-care molecular test using isothermal nucleic acid amplification technology (NAAT) for detecting nucleic acid from SARS CoV-2. The analysis time on the instrument was 13 minutes (Barnacle et al., 2022; Dinnes et al., 2020). The Rida gene RT-PCR assay uses internal control RNA to control for PCR inhibition in each sample. The DirectDetect SARS-CoV-2 assay uses oligonucleotides to amplify RNA from the human 16s rRNA gene as internal control to ensure that cellular material is investigated, validating sample collection, transport, RNA release, and RT-PCR inhibition. Both kits require positive internal control amplification for negative results, as well as samples in batches of up to 94 and for every batch run, using positive and negative controls for approval of each run. Additionally, a pre-prepared positive and negative sample was included in each batch to control the workflow. The ID NOW assay analyzes samples individually, using an endogenous internal control to validate negative results. Each new batch of kits is approved using a kit positive and negative control. Positive and negative controls are not used for patient samples, as the manufacturer instructs.

### Serological tests

After 2 years of pandemic, just before ending the testing regime and IPC measures at the workplace, participating laboratory staff were offered a blood test and return a questionnaire. SARS-CoV-2 IgG II Quant and SARS-CoV-2 IgM Architect from Abbott Norge AS were used to detect spike IgG and IgM. Elecsys Anti-SARS-CoV-2 (IgM + IgG) on a Cobas e411 from Roche Diagnostics AS (Oslo, Norway) was used to detect SARS-CoV-2 nucleocapsid total antibodies. For the Internal Quality Control (IQC), both kit-dependent controls (CoV-2 IgM Ctrls, CoV-2 IgG II Ctrls, PreciControl Anti-SARS-CoV-2) and local kit-independent controls were used. Independent control kit was used to evaluate precision. The mean values for SARS-CoV-2 IgM and IgG were 1.733 and 72.1, with coefficients of variation (CV) of 2.2 and 3.1. For IgG nucleocapsid, the mean value was 3.29, with a CV of 4.3. The laboratory participated in the External Quality Control (EQC) schemes UK Neqas Immunology CoV-2 (2020-2021) and UK Neqas serology SARS-CoV-2 (2022), both with satisfactory results.

### Questionnaire and sick leave statistics

All participants received an Self-administered questionnaire detailing their COVID-19 history (Table 1), linked with a serum sample for SARS-CoV-2 serology with a barcode. Anonymized sickness absence data was extracted from local human resources systems and comparative analyses were conducted between the pre-pandemic year (2019) and pandemic years (2020 and 2021).

**Table 1.** Self-administered questionnaire.

Covid-19		
1.	Have you had covid 19 which has been confirmed by PCR? - If yes, when did you test positive? - Where/by whom do you assume you were infected? - Do you suspect that you have infected others at work? - Have you had symptoms of long covid?	Yes/No dd.mm.yy Colleague/household/Friend/acquaintance/Patient contact/Abroad/Cultural events/Don’t know/Other Yes/No Yes/No
2.	Have you had respiratory symptoms and tested negative with a PCR? - If so; could you return to work after a negative test result?	Yes/No Yes/No
3.	Have you been a close contact and tested negative with PCR or rapid PCR?	Yes/No
Health information		
4.	Do you have an underlying disease that defines you in the risk group?	Yes/No
5.	Are you vaccinated? - If so; How many doses?	Yes/No Number
6.	Do you take supplements of vitamin D, cod liver oil and/or vitamin C?	Yes/No
7.	Are you immunosuppressed?	Yes/No
Other		
8.	Have you mainly used public transit for work during the pandemic?	Yes/No
9.	Do you have children who go to kindergarten or school?	Yes/No
10.	Is your place of work at one of the sampling stations	Yes/No
11.	Have you been absent from work more than 2 months continuously during the period?	Yes/No
12.	Age	0–20/21–40/41–60/61–80/80+
13.	Gender	Male/Female

### Statistical analysis

Statistical analysis was performed and graphs rendered in R 4.3.1. (R Core Team, 2023) using RStudio 2023.9.1.494 (Posit team, 2023). Confidence intervals for workplace sickness absence at Fürst Medical Laboratory were calculated using the binomial distribution.

## Results

Of the 153 participating employees in the study, 69 (45%) reported not having had a COVID-19 infection, while 84 (55%) indicated having tested positive for SARS-CoV-2 during the study period. The self-reported route of transmission from those 84 (55%) individuals is summarized in Table 2. The most common place to contract COVID-19 was in the household, which was reported by 51% of respondents knowing their probable route of transmission.

**Table 2.** Self-reported route of COVID-19 contraction.

Route	*N*	%	% of known
Colleague	12	14.3	20.3
Household	30	35.7	50.8
Friend/acquaintance	7	8.3	11.9
Foreign country	1	1.2	1.7
Cultural events	3	3.6	5.1
Patient contact	1	1.2	1.7
Other	5	6	8.5
Don’t know	24	28.6	
Not stated	1	1.2	
Total	84		

Furthermore, we investigated how many of the employees had experienced respiratory symptoms yet tested negative for SARS-CoV-2. One hundred and one (66%) had experienced symptoms followed by a negative test. Forty-nine employees (32%) reported either a positive test or not having experienced respiratory symptoms, and 3 employees (2%) did not provide this information.

Out of 104 employees tested with PCR-based tests for SARS-CoV-2, 80 (79%) had only negative results, and 24 (23%) had at least one positive molecular SARS-CoV-2 test during the test period.

Sixty-nine employees reported not having undergone a COVID-19 infection. Serology results confirmed SARS-CoV-2 vaccination in 90% of participants. In 8.7% tests indicated previous COVID-19 infection, and 1.3% suggested recent vaccination or recent COVID-19 infection (Table 3).

**Table 3.** SARS-CoV-2 serology in employees without reported COVID-19 infection.

SARS-CoV-2 spike IgM	SARS-CoV-2 spike IgG	SARS-CoV-2 nucleocapsid (IgM + IgG)	*N* = 69
NEG	POS	NEG	62 (90%)
NEG	POS	POS	6 (8.7%)
POS	POS	NEG	1 (1.3%)

**Figure 1.**
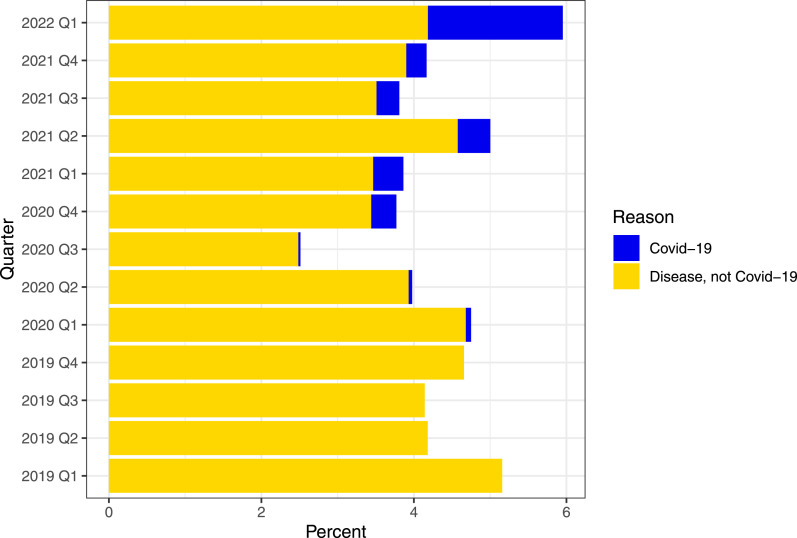
Aggregated quarterly workplace sickness absence at the medical laboratory. Statistics is presented as percent from total number of employees (N=437). Workplace sickness absence is grouped in covid-19 and non-covid-19.

Figure 1 shows aggregated workplace sickness absence statistics for all employees at Fürst medical laboratory from 2019 to Q1 2022, grouped into COVID-19 and non-COVID-19. Mean sickness absence during the pandemic (2020: 3.74%, 2021: 4.19%) was significantly lower compared to pre-pandemic 2019 (4.54%, *p* < .001).

## Discussion

This project aimed to assess whether IPC and accessible SARS-CoV-2 PCR testing prevented internal spread of infection in the workplace and minimized absences by quickly identifying uninfected individuals who were in good general health and thereby could return to work. Effective IPC, based on the national recommendations, were important to prevent internal spread of infection at the workplace. The employees who had a negative SARS-CoV-2 test and good general health condition could return to work within 1 hour to a day. Most received results within 30 minutes (ID NOW™) to a few hours (DirectDetect™ SARS-CoV-2). Confirmed cases were isolated according to applicable guidelines. Antigen tests were not used to rule out infection as the sensitivity was perceived as low and uncertain considering the asymptomatic and those with mild symptoms (Dinnes et al., 2020; Jegerlehner et al., 2021; Lippi et al., 2020).

### COVID-19 infection and route of transmission

Twelve employees (14.3%) testing positive for SARS-CoV-2, suspected transmission from a colleague. Testing was offered daily to symptomatic employees. The relatively low number of suspected colleague transmissions may indicate that infection control routines have worked well. However, it is important to point out that the vaccination rate in the population was high and we did not have the opportunity to include a control group. Additionally, 21% (*N* = 25) were unsure or did not state the transmission route. The limitation of a questionnaire is that it emphasizes the participant’s subjective perception of the infection route, as this study did not offer the opportunity to identify an objectively verified transmission pathway.

A case-control study by The Norwegian Institute of Public Health in 2021 found that 11% of the population stated the route of infection from the workplace, with increased risk when remote work was not possible (Stålcrantz et al., 2021). A Danish study showed that 30% stated workplace as the site of infection and that having many close contacts in the workplace was a risk factor (Munch et al., 2022). Our population differs as participants mostly did not work from home and they were living in or around Oslo, as Oslo had a higher rate of transmission of COVID-19 than Denmark and Norway in general. The potential transmission rate of 14% observed at Fürst Medical Laboratory can thus be considered relatively low, but no control group has been studied.

### Asymptomatic COVID-19 infection

Serum samples from employees were analyzed for SARS-CoV-2 spike IgG, spike IgM, and nucleocapsid IgG at Fürst Medical Laboratory (Table 3). The spike protein is present in both vaccine and virus, so it cannot distinguish between vaccination and infection. The nucleocapsid protein is only found in the virus. Nucleocapsid IgG is therefore only positive in those infected with SARS-CoV-2 allowing detection of previous asymptomatic COVID-19 (Amanat et al., 2020; Lippi et al., 2020; Mariën et al., 2021).

Among employees who reported not having been infected by SARS-CoV-2, 8.7% (*N* = 6) tested positive for SARS-CoV-2 IgG nucleocapsid, indicating virus exposure. This group was not detected by the test procedure at the time. The potential presence of asymptomatic individuals in the laboratory stresses the importance of IPC, as they may pose a transmission risk. A previous study (Kronbichler et al., 2020) found that almost all asymptomatic cases were detected through screening of close contacts. During high transmission periods, prioritizing testing of close contacts and symptomatic individuals while maintaining IPC could be reasonable.

### Entering the workplace after a negative test for COVID-19

66% (*N* = 101) of employees experienced respiratory symptoms and subsequently tested negative for SARS-CoV-2. Of these, 79% (*N* = 80) were in good general health condition and could enter the workplace and follow the IPC that had been implemented. In general, people with pronounced respiratory symptoms were not allowed to enter the workplace regardless of the test result, as it was recommended to reduce transmission of non-COVID-19 respiratory diseases in society. It was not guaranteed that individuals testing negative initially would not test positive later, neither for SARS-CoV-2 nor for non-COVID-19 agents, and thus would not be detected by the outlined testing regime. The laboratory had a high testing workload throughout the pandemic and ensuring a 79% return to work was important to uphold the laboratory’s essential societal task.

### Shorter time to test results ensures more time at the workplace

The fast-track testing regime potentially added 1–3 workdays for 79% of employees (80 out of 104) during the 25 months from the pandemic onset until the conclusion of IPC in April 2022. Absences would likely have been more frequent during high transmission periods coupled with a particularly high laboratory workload.

Local medical leave data (Figure 1) shows lower sickness absence during the pandemic years (2020 and 2021) compared to the pre-pandemic year (2019). One can suspect that sickness absence may rise during periods of widespread illness within the community, but that the implementation of effective IPC and extensive testing have contributed to keeping sickness absence somewhat lower. It is also important to emphasize that the employees were impacted by national IPC, which in turn influenced these findings.

In epidemics and pandemics, established IPC measures are essential to prevent spread. Based on the material available in this project, it appears that pronounced internal transmission among the employees has been avoided. One of the reasons may be the use of established infection control routines in place. It seems that by having frequent, accessible and accurate, rapid molecular testing for SARS-CoV-2, one has managed to maintain necessary personnel. In the larger perspective as well, it can be argued that easy access to a fast-track rapid molecular testing regime could be feasible in other settings (clearing work force in hospital/visitors in hospitals/other public institutions a. o). It is therefore reasonable to believe that it would be appropriate to focus on having readily available testing strategies, necessary resources, and procedures for how to approach new pandemics and epidemics in the future.
